# Mental health symptoms and everyday social interaction among university students: an exploratory intensive longitudinal study of relational events within bounded peer networks

**DOI:** 10.1186/s40359-026-05805-7

**Published:** 2026-10-15

**Authors:** Heike Krüger

**Affiliations:** 1 Institute of Sociology, RWTH Aachen University, Eilfschornsteinstr. 7, 52062 Aachen, Germany; 2 Department of Sociology and Social Psychology, University of Cologne, Cologne, Germany

**Keywords:** Mental health, University students, Ecological momentary assessment, Social interaction, Emotional support, Social networks, Dynamic Network Actor Models (DyNAMs)

## Abstract

**Background:**

Mental health symptoms are closely linked to social relationships, yet most research examines social connectedness through stable ties or retrospective reports. Less is known about how symptoms are associated with the unfolding micro-dynamics of everyday interaction. This exploratory study examined whether depressive symptoms, anxiety symptoms, and ADHD symptoms were differentially associated with face-to-face contact and received emotional support among university students.

**Methods:**

Data came from the PeerSmart Network Study, an intensive longitudinal social network study combining smartphone-based ecological momentary assessment with roster-based sociometric measurement. Thirty-eight students from two bounded academic cohorts completed baseline mental health measures and participated in a seven-day assessment period with three prompts per day, contributing 657 short surveys. At each prompt, participants reported face-to-face contacts lasting at least five minutes and received emotional support since the previous survey. Given the intensive but small-scale design, the analyses were intended to model micro-level interaction processes within bounded peer contexts rather than to estimate population-level associations. Dynamic Network Actor Models were estimated separately for the bachelor and master cohorts. Received emotional support was modelled as a directed event process, whereas face-to-face contact was modelled as an undirected coordination process.

**Results:**

Mental health symptom levels were more consistently associated with received emotional support than with general face-to-face contact. Anxiety symptoms showed a positive association with receiving emotional support in both cohorts. Depressive symptoms were negatively associated with receiving emotional support in the bachelor cohort, and ADHD symptoms were negatively associated with receiving support in both cohorts. In contrast, face-to-face contact showed little evidence of overall involvement differences by symptom level, suggesting that general social exposure was more strongly associated with prior interaction history and opportunity structures.

**Conclusions:**

The findings provide preliminary evidence that mental health symptoms are not uniformly associated with everyday social interaction. Rather, symptom levels appeared to be most relevant for emotionally meaningful support exchanges. These findings highlight the importance of distinguishing general social contact from support-related interaction and suggest that student mental health interventions should attend not only to social connectedness, but also to the concrete contexts in which support is mobilized or missed.

**Supplementary Information:**

The online version contains supplementary material available at https://doi.org/10.1186/s40359-026-05805-7.

## Introduction

Mental health is deeply embedded in everyday social life. Extensive previous research demonstrates robust associations between mental health and social connectedness, showing that depressive and anxiety symptoms are linked to fewer social contacts, lower perceived support, and heightened loneliness [1–4]. Yet, despite decades of work on social relationships and health, most empirical studies conceptualize social relations as relatively stable structures such as yearly measured friendship ties [5] or perceived availability of support [6] rather than as sequences of concrete social actions unfolding in time. This static perspective obscures the micro-level processes through which mental health shapes, and is shaped by, everyday interaction.

From a processual standpoint, social interaction consists of moment-to-moment decisions: whether to approach someone, whether to respond, whether to disclose distress, or whether to provide emotional support. These decisions are embedded in specific situations and influenced by affective states. Relational theories have long emphasized that social structure emerges from repeated, situated actions rather than from fixed ties [7]. In line with this view, social relationships should be understood as outcomes of accumulated interaction events rather than as static entities that exist independently of ongoing social practice.

If social interaction is understood as a sequence of situated decisions, then mental health symptoms are likely to influence these decisions through distinct psychological and behavioural pathways. Different forms of psychopathology shape how individuals perceive social situations, regulate affect, and coordinate behaviour during interaction. In this study we focus on depressive symptoms, anxiety symptoms, and ADHD symptoms because these symptom domains capture three theoretical mechanisms that are particularly relevant for everyday interaction processes. Depressive symptoms are closely linked to reduced approach motivation, altered affective engagement, and negatively biased interpretations of interpersonal feedback [8, 9]. Anxiety symptoms primarily affect perceptions of social threat and evaluation, which can alter the willingness to engage with others and increase reliance on interactions perceived as safe [10]. ADHD symptoms, in contrast, influence interaction through difficulties in attentional control and impulse regulation, which affect the coordination of conversational exchange and the interpretation of social cues [11, 12]. These symptom profiles therefore represent complementary mechanisms through which mental health may shape the micro-dynamics of everyday social interaction. Examining these mechanisms jointly allows us to assess whether different dimensions of mental health systematically influence how individuals initiate contact, respond to others, and exchange emotional support in daily life.

Depression and anxiety show substantial overlap and are commonly understood as closely related manifestations of broader internalizing psychopathology [13]. ADHD is likewise frequently accompanied by anxiety and depressive symptoms; a recent meta-analytic review of large adult samples found markedly elevated rates of both anxiety disorders and major depression among adults with ADHD [14]. More broadly, transdiagnostic research suggests that conventional psychopathology domains partly reflect shared underlying liabilities rather than fully separable processes [13]. Accordingly, the mechanisms discussed below should be understood as partly overlapping rather than disorder-specific. Distinguishing the symptom domains nevertheless remains useful for examining whether they show different patterns of association with everyday social interaction.

### Depressive symptoms and patterns of social engagement

In the case of depressive symptoms, longitudinal network research suggests reciprocal links between mental health and social relationships. Depressive symptoms have been associated with subsequent friendship withdrawal and exclusion [1, 2]. Elmer et al. [15] likewise found evidence for selection from emotional well-being to friendship development. Beyond reduced activity, depressive symptoms have been associated with observable changes in nonverbal behaviour. In a longitudinal study following depressed individuals over the course of treatment, greater symptom severity was associated with less smiling and more contempt-related facial expressions, with these patterns diminishing as symptoms improved [16]. Cognitive models of depression further suggest that negatively biased interpretations of social feedback lead individuals to discount positive interactions and perceive ambiguous or even supportive responses as rejection or indifference [8, 17]. These cognitive distortions weaken the reinforcing effects of social interaction and reduce the likelihood that prior positive experiences translate into future approach behaviour.

At the same time, depressive symptoms may increase reassurance-seeking and emotional support needs, particularly under stress [18]. This creates a paradoxical dynamic: individuals may seek more emotional support while simultaneously engaging in interaction styles that strain relationships. Interpersonal models of depression propose that persistent reassurance seeking and negatively toned interaction patterns may initially elicit responses from others but, over time, contribute to rejection and relationship strain [9, 18]. From a dynamic interaction perspective, this implies that depressive symptoms can simultaneously elevate the likelihood of support-seeking events while reducing the probability of sustained reciprocal exchange over time, producing unstable or self-reinforcing interaction trajectories.

### Anxiety and the selectivity of social interaction

Anxiety-related symptoms shape interaction processes through mechanisms that are partly distinct from depression and especially consequential for the initiation and selectivity of social engagement. Interpersonal and cognitive models of anxiety emphasize heightened threat monitoring, fear of negative evaluation, and the use of safety-oriented behavioural strategies, all of which can inhibit spontaneous approach behaviour and make social participation more contingent on perceived interpersonal safety [10]. Rather than uniformly reducing sociality, anxiety may reorganize it: anxious individuals may avoid unfamiliar, evaluative, or demanding encounters while concentrating interaction within a smaller set of trusted partners. Cross-sectional, multi-informant research is consistent with anxiety-related differences in interpersonal functioning. Social anxiety has been associated with self-reported social avoidance and reduced interpersonal assertiveness, while correspondence with informant reports is more limited [19, 20]. Laboratory-based observational evidence further suggests that social anxiety is associated with specific aspects of social performance. Among university students completing standardized interaction and speech tasks, higher social anxiety was associated with greater observer-rated behavioural discomfort [21].

These dynamics may operate differently for emotional support exchange. Anxiety symptoms have been associated with excessive reassurance seeking [22], which may increase the likelihood that anxious individuals receive emotional support in everyday life. Notably, longitudinal evidence also suggests that this relationship may operate in the opposite direction, with excessive reassurance seeking predicting subsequent increases in social anxiety and generalized anxiety symptoms [22]. In a laboratory study individuals high in social anxiety showed a greater tendency to interpret others’ emotional expressions as negative, without showing superior discrimination of emotional states [23], which may make the disclosure of distress socially demanding. Rather than implying a straightforward withdrawal from social interaction, anxiety symptoms may therefore be associated with a greater need for reassurance and emotional support. From a dynamic interaction perspective, anxiety symptoms are thus expected to increase the likelihood of receiving emotional support, even if their association with general face-to-face contact remains less clear.

### ADHD and the coordination of social interaction

ADHD-related symptoms influence social interaction through mechanisms that operate directly at the level of interaction coordination. Core features of ADHD, including inattention, impulsivity, and difficulties in executive control, can disrupt the perception and interpretation of social cues that guide moment-to-moment interaction. Individuals with elevated ADHD symptoms are more likely to miss subtle signals such as conversational timing, facial expressions, or shifts in group attention, which increases the risk of misaligned responses and interaction breakdowns [12]. Impulsivity further affects conversational mechanics by increasing interruptions, topic shifts, and difficulties regulating turn-taking, behaviours that can strain peer relationships even when social motivation remains high [11, 24]. These micro-level interaction difficulties accumulate into broader relational consequences. A review of the literature on peer functioning concluded that ADHD is consistently associated with problematic peer relationships, including rejection and interpersonal difficulties [25]. As a result, interaction opportunities may become less predictable and less reciprocally sustained over time. These dynamics also have implications for emotional support processes. Providing and mobilizing support typically requires sustained attention to others’ emotional signals and coordinated conversational exchange. Difficulties in attentional control and impulse regulation can therefore hinder both the provision and receipt of support. From a dynamic interaction perspective, ADHD symptoms are thus expected to negatively influence the likelihood of initiating interaction.

Taken together, these theoretical perspectives suggest that mental health symptoms may shape everyday social life through subtle but consequential differences in how interactions are initiated. However, empirical research has struggled to capture these processes because most available data rely on retrospective and aggregated reports of social relationships. A growing number of studies have begun to address this limitation using passive sensing technologies such as RFID tags to record face-to-face encounters in real time. A prospective observational study of university students during an orientation weekend found that individuals with higher depressive symptoms spent less time interacting overall and were more likely to interact with similarly depressed peers [26]. Yet findings from a three-month longitudinal RFID study of early adolescents were more mixed: using proximity data in school cafeterias, the longitudinal network models did not provide corresponding evidence that depressive symptoms shaped the development of interaction ties [27]. While such sensor-based approaches provide valuable insights into the spatial structure of interaction opportunities, they are typically limited to specific physical environments and capture only co-presence rather than the social meaning of interaction. As a result, they cannot distinguish between different types of interaction, such as casual contact versus emotionally supportive exchange.

### Studying everyday interaction within bounded social contexts

Traditional EMA approaches to social interaction often prioritize ecological breadth by allowing respondents to report encounters across different domains of everyday life and with potentially unrestricted interaction partners. This provides valuable information about individuals’ overall social experiences, but makes it more difficult to locate individual encounters within a clearly defined relational system. Bounded interaction designs offer a complementary perspective by specifying, a priori, a researcher-defined set of actors who constitute the social context under study. This boundary defines the set of eligible interaction partners against which observed interaction choices can be evaluated. Consequently, it can not only be examined who interacted with whom but also whether the characteristics of actors and dyads involved in observed interactions differ systematically from those of eligible alternatives within the same relational context. In this sense, non-occurring interactions are also analytically informative because observed events can be evaluated relative to a defined set of possible interaction partners or dyads. Repeated observations within the same bounded system further make it possible to assess how current interactions depend on previous dyadic interaction histories and on the evolving relational structure, including processes such as repeated interaction, reciprocity, and local clustering [28, 29]. Accounting for these endogenous dependencies is important because current interaction opportunities are not independent of previous encounters or the surrounding relational structure; modelling them therefore helps distinguish associations with individual or dyadic characteristics from the tendency of interaction patterns to reproduce and cluster over time.

For research on mental health and social relationships, this relational perspective is particularly relevant because differences associated with mental health symptoms may concern not only overall levels of social contact, but also individuals’ positions within recurring patterns of interaction and support [26, 27]. The trade-off is a narrower representation of social life: bounded designs characterize interaction within a theoretically defined social setting rather than across an individual’s entire personal network.

### The present study

Building on the bounded relational perspective, the present study combines smartphone-based experience sampling with cohort-specific sociometric rosters to capture everyday interaction processes within clearly defined university peer contexts. The roster-based design links each reported interaction to an identifiable dyad and defines the set of eligible cohort members against which observed interaction patterns can be evaluated. First, rather than focusing on the presence or absence of social ties, we conceptualize social interaction as a sequence of discrete events unfolding over time and examine how mental health symptoms are associated with involvement in these interaction processes. Although prior longitudinal research points to potentially reciprocal links between mental health and social relationships, the present study focuses specifically on associations between baseline mental health symptoms and subsequent interaction events. Second, we distinguish between two substantively different forms of interaction, face-to-face contact and emotional social support exchange, allowing us to assess whether associations with mental health symptoms differ between general social exposure and support-related interaction. Third, recognizing that depressive symptoms, anxiety symptoms, and ADHD symptoms are interrelated, we consider all three dimensions simultaneously to examine whether each shows a distinct conditional pattern of association with everyday interaction when the other symptom dimensions are taken into account. Finally, we analyse these processes using Dynamic Network Actor Models (DyNAMs), which allow us to examine associations between mental health symptoms and event rates, partner selection, and symptom similarity while accounting for endogenous dependencies arising from prior interaction histories, reciprocity, and local clustering [30].

The study further draws on two academic cohorts that represent distinct stages of peer-network development: first-semester bachelor students entering a newly forming academic environment and master students embedded in a more established university peer context. Entering university typically involves the formation of a new social environment in which peer relations develop rapidly and early interaction opportunities can have lasting consequences for subsequent network structure (31). Longitudinal network research shows that these networks develop substantially over the first academic year, with early contact opportunities shaping friendship formation and the broader peer network becoming increasingly structured over time [31, 32]. The first-semester bachelor cohort in the present study therefore represents a comparatively newly forming peer context in which many relationships and recurring interaction patterns are still being established. The master cohort, in contrast, includes students across semesters one to six and thus represents a socially more heterogeneous context in terms of prior exposure to the academic environment and accumulated interaction histories. We therefore treat the two cohorts not as a direct developmental comparison between bachelor and master students, but as distinct relational contexts in which the association between mental health symptoms and everyday interaction may unfold against different degrees of pre-existing social structure.

By linking variation in mental health symptoms to the unfolding dynamics of everyday social interaction, the study provides exploratory intensive longitudinal evidence on how social relationships are enacted in daily life. Rather than estimating generalizable population-level associations, the study is designed to model micro-level interaction processes within bounded university peer contexts.

## Methods

### Data

Data were drawn from the PeerSmart Network Study, an intensive longitudinal social network study. The study combined smartphone-based ecological momentary assessment (EMA) with roster-based sociometric network measurement. The study population consists of bachelor’s and master’s students enrolled in social science programs at a large German university. Eligibility was defined separately for two student groups. The first group comprised first-semester bachelor students enrolled in the respective social science degree programme, representing a newly formed academic and social environment. The second group comprised master’s students enrolled in the same degree programme across semesters one to six, representing a more established but still clearly delimited academic peer setting. All students belonging to one of these two groups were eligible to participate and were invited to the study via their university email accounts. In addition, the study was advertised in lectures and seminars attended by the target populations. Data collection for the bachelor cohort took place in late January and early February 2025, whereas the master cohort was surveyed in May 2025.

In total, 38 individuals participated in the study. The bachelor cohort included 23 students (participation rate: 41% of students enrolled in the programme), and the master cohort included 15 students (participation rate: 29% of students enrolled in the programme). With regard to gender, the bachelor cohort comprised 17 women (73.9%), five men (21.7%), and one participant who identified as diverse (4.3%). The master cohort comprised seven women (46.7%), seven men (46.7%), and one participant who identified as diverse (6.7%). The age distribution reflected the different educational stages of the two cohorts. In the bachelor cohort, 30.4% of participants were aged 18 or 19 years, 39.1% were aged 20 or 21 years, and 30.4% were aged 22 years or older. In the master cohort, 46.7% of participants were aged 22 to 25 years, 33.3% were aged 26 or 27 years, and 20.0% were aged 28 years or older. Participants also reported indicators of their socioeconomic background. Parental education was assessed as the highest educational qualification attained by at least one parent. In the bachelor cohort, 43.5% reported that at least one parent held a university degree, 21.7% reported a university entrance qualification, 30.4% an intermediate secondary school qualification, and 4.3% a lower secondary school qualification. The corresponding proportions in the master cohort were 46.7%, 26.7%, 20.0%, and 6.7%, respectively. Participants additionally rated their family’s financial position within German society on a 10-point scale ranging from 1 (wealthiest families) to 10 (financially worst-off families). Mean ratings were 4.52 (range = 1–8) in the bachelor cohort and 5.47 (range = 3–10) in the master cohort.

Across the seven-day ecological momentary assessment period, participants completed a total of 657 short surveys out of 798 possible surveys, corresponding to an overall compliance rate of 82.3%. Bachelor students contributed 371 surveys (mean = 16.1 per person of 21 surveys; compliance rate = 76.8%), and master students contributed 286 surveys (mean = 19.1 per person of 21 surveys; compliance rate = 90.8%).

### Procedure

Prior to the intensive data collection phase, participants completed an online baseline survey assessing mental health and other stable characteristics. The baseline survey had to be completed between 14 days and 1 day before the start of the EMA period. Within each cohort, all participants entered the seven-day ecological momentary assessment period simultaneously. This cohort-level synchronization ensured that interaction reports referred to the same observation window.

During the EMA period, participants received three smartphone-based short surveys per day at fixed times (12:00, 17:00, and 22:00). Participants had a response window of 60 min to complete each survey. Data collection took place during the university lecture period. The timing of the midday and afternoon prompts was deliberately aligned with the end of common university teaching slots to increase the likelihood that participants had recently engaged in social interaction and were available to respond shortly after classes or seminars had ended. The surveys assessed social interactions that had occurred since the previous prompt. Participants were instructed to respond as promptly as possible and to base their reports on concrete interaction episodes rather than general impressions.

Participants received financial and material incentives for participation. Completion of the baseline survey was compensated with a €5 Amazon voucher. Participants who completed at least 16 short surveys received an additional €30 Amazon voucher, whereas participants completing fewer than 16 short surveys received an additional €10 voucher. In addition, participants were allowed to keep the Fitbit Inspire 3 device used during the broader study after study completion. The wearable devices were included to collect physiological and behavioural data for other project objectives not examined in the present analyses.

The study protocol was approved by the Interfaculty Ethics Committee of RWTH Aachen University (Reference Number FÜEK: 30/24), and all participants provided informed consent prior to participation.

### Measures

#### Personal face-to-face interaction

At each EMA prompt, participants reported whether they had engaged in any personal face-to-face contact lasting at least five minutes since the previous survey. Face-to-face interaction was defined as in-person social contact involving direct interpersonal engagement, excluding purely digital communication. For each reported contact, participants selected the interaction partner(s) from a predefined roster containing all students enrolled within their respective cohort boundaries, including students who did not participate in the study themselves. The rosters were constructed using official university enrolment lists. To reduce identifiability and protect privacy, roster entries were limited to first names and the first two letters of surnames.

For bachelor students, nominations were restricted to students enrolled in the first semester of the respective degree programme. For master students, nominations were restricted to students enrolled in the same degree programme between the first and sixth semester. Individuals outside these cohort boundaries could not be nominated. Participants could, however, nominate enrolled cohort members irrespective of study participation status.

Each reported contact generated an undirected interaction event between the focal participant and the nominated interaction partner. Because face-to-face encounters are inherently reciprocal and the reporting participant cannot necessarily be interpreted as the initiator of the interaction, personal contacts were operationalized as symmetric dyadic events. Events were time-stamped at the level of the reporting interval.

#### Received emotional social support

At each EMA prompt, participants were asked to indicate with whom they had discussed or exchanged about their own personal problems or something that was troubling them since the previous survey. When support was reported, participants identified the provider(s) of support using the same roster-based selection procedure described above. The item explicitly noted that such exchanges could, for example, take place in person or online; the specific communication mode was not otherwise assessed. In contrast to the face-to-face contact measure, no minimum duration was specified for emotional support interactions. Thus, the two interaction measures capture substantively different forms of social exchange: face-to-face contact reflects in-person interaction lasting at least five minutes, whereas emotional support captures problem-focused supportive exchange irrespective of communication mode or duration.

In contrast to face-to-face contact, emotional support exchange was operationalized as a directed interaction process because support interactions imply asymmetric provider–receiver roles. Each reported support exchange therefore generated a directed event from the support receiver (sender) to the participant providing support (receiver).

#### Depression

Depressive symptoms were assessed using the Patient Health Questionnaire (PHQ-9) [33], a nine-item self-report instrument measuring the frequency of core depressive symptoms over the past two weeks. The PHQ-9 operationalizes depression in line with DSM diagnostic criteria and captures affective, cognitive, and somatic symptom dimensions, including anhedonia, depressed mood, sleep problems, fatigue, and concentration difficulties. Items are rated from 0 (“not at all”) to 3 (“nearly every day”), yielding a total score ranging from 0 to 27. Higher scores indicate greater depressive symptom severity. In the analyses, depressive symptoms were treated as a continuous symptom score. Internal consistency was acceptable in both cohorts, with Cronbach’s alpha values of 0.75 in the bachelor cohort and 0.78 in the master cohort.

#### Anxiety

Generalized anxiety symptoms were measured using the Generalized Anxiety Disorder scale (GAD-7) [34]. The GAD-7 is a seven-item instrument assessing the frequency of key anxiety symptoms over the past two weeks, including excessive worry, nervousness, restlessness, and difficulty relaxing. Items are rated from 0 (“not at all”) to 3 (“nearly every day”), resulting in a total score ranging from 0 to 21. Higher scores indicate greater generalized anxiety symptom severity. In the analyses, anxiety was treated as a continuous symptom score. Internal consistency was acceptable to good, with Cronbach’s alpha values of 0.76 in the bachelor cohort and 0.87 in the master cohort.

#### ADHD

ADHD symptoms were assessed using the Adult ADHD Self-Report Scale screening scale (ASRS v1.1) [35]. The ASRS is a validated self-report measure designed to capture core symptoms of attention-deficit/hyperactivity disorder in adults, covering both inattention and hyperactivity–impulsivity. We followed the updated scoring approach recommended for research on ADHD prevalence and correlates [36]. Responses are coded from 0 (“never”) to 4 (“very often”) and summed across the six items, yielding a total score ranging from 0 to 24. Higher scores indicate more frequent ADHD symptoms. Internal consistency differed across cohorts, with a Cronbach’s alpha of 0.78 in the master cohort and a lower value of 0.55 in the bachelor cohort. The ADHD results should therefore be interpreted with caution in the bachelor cohort.

#### Control variables

The models additionally include a set of actor-level and endogenous network control variables to account for established regularities in social interaction and support exchange. First, gender was included as an actor attribute at both the sender and receiver level. Prior research consistently shows that women report higher prevalence rates of depressive and anxiety symptoms and are more likely to engage in emotional support provision and supportive communication [4]. Moreover, social interaction and support exchange frequently exhibit gender homophily, such that interactions between individuals of the same gender occur more often than expected by chance [37]. To account for these tendencies, the models include ego gender effects, alter gender effects, and same-gender similarity effects.

In addition, the models include endogenous network effects capturing temporal dependencies in interaction sequences. Inertia models the tendency for previously occurring interaction patterns to persist over time, reflecting the fact that individuals are more likely to interact repeatedly with familiar partners. Reciprocity captures the tendency for interactions to be returned by interaction partners. Reciprocity is particularly relevant for emotional support exchange, where supportive behaviour often generates expectations of mutual responsiveness and relational obligation [38]. Finally, transitivity captures tendencies toward locally clustered interaction structures, where interaction becomes more likely between individuals connected through shared partners [39]. Including these endogenous effects is important to distinguish associations with mental health symptoms from structural regularities inherent to evolving interaction networks.

### Analytical strategy

The analyses are based on relational event modelling, a class of statistical models designed to study sequences of (social) interactions unfolding over time [28]. In contrast to network models that focus on the presence or strength of enduring ties, relational event models conceptualize interactions as a series of discrete, time-stamped events. Each event represents an interaction occurring at a specific point in time and is modelled conditional on actor characteristics, dyadic attributes, and the history of previous interactions. This framework is particularly well suited for studying social interaction as an ongoing process rather than as a static relational structure.

To model these processes, we employ Dynamic Network Actor Models (DyNAMs) [29, 40], an actor-oriented extension of the relational event framework. DyNAMs conceptualize interactions as purposeful social actions embedded in evolving interaction histories. Depending on the nature of the interaction process, DyNAMs can be specified for directed interaction events or for undirected coordination events [30].

Formally, DyNAMs are formulated in terms of event rates or hazards, describing the instantaneous propensity for an interaction event to occur conditional on the current state of the interaction process. For directed relational events, the actor-oriented DyNAM decomposes this process into two components. The rate component models actors’ relative propensity to become active in the next event, whereas the choice component models the conditional probability of selecting a particular interaction partner from the set of eligible alters once an actor becomes active [29]. This separation makes it possible to distinguish characteristics associated with actors’ general involvement in the event process from characteristics associated with the relational patterning of interaction partners. Both components can additionally depend on the evolving interaction history and other actor- or dyad-level characteristics.

For received emotional social support, we estimated directed DyNAMs because support exchange implies asymmetric provider–receiver roles. Directed DyNAMs model interaction as a two-stage process in which actors first become active and subsequently select a specific interaction partner from the set of available alters [29]. Accordingly, the support models consist of (a) a rate component capturing the propensity of actors to become involved in support-related interaction events and (b) a choice component modelling which interaction partner is selected conditional on an event occurring. This specification allows support exchange to be analysed as an explicitly directional social process.

In contrast, personal face-to-face contact was modelled using undirected coordination DyNAMs [30]. Face-to-face encounters cannot meaningfully be reduced to unilateral initiation by a single actor. Rather than modelling sender-to-receiver actions, coordination models conceptualize interaction as the formation of a temporary undirected interaction event between two actors. This approach is particularly appropriate for everyday co-present interaction, where social encounters emerge through mutual coordination and shared situational opportunities rather than strictly directional action.

Although interaction reports were collected within discrete EMA reporting intervals rather than as exact continuous-time observations, DyNAM estimation requires temporally ordered event sequences with unique timestamps. To approximate the temporal ordering of interaction events, event times were therefore operationalized using the exact submission timestamp of the corresponding EMA survey rather than the nominal survey interval itself. When participants reported multiple interaction events within the same survey, these events initially shared identical timestamps. To satisfy the ordering assumptions of relational event models and avoid tied event times, simultaneous events were minimally jittered by adding microsecond-level random offsets to events occurring within the same submission timestamp. Additional minimal offsets of a few seconds were applied where necessary to ensure global uniqueness of timestamps across all events [41]. These adjustments preserved the observed ordering of reporting windows while introducing only negligible perturbations to the temporal structure of the data. The resulting event streams therefore approximate continuous-time interaction sequence.

In the EMA surveys, participants were able to nominate interaction partners outside the analytically defined study sample, provided these individuals fell within the broader cohort rosters. However, DyNAM estimation requires observed actor attributes for both individuals involved in an interaction event. Consequently, only interaction events between two study participants could be included in the analytical models. Interactions involving non-participating cohort members were excluded because no corresponding mental health measures or actor-level covariates were available for these alters. The proportion of interaction nominations retained for analysis differed across cohorts and interaction types. In the bachelor cohort, 84.7% of face-to-face contact nominations and 90.9% of received emotional support nominations involved participating students and could therefore be included in the DyNAM analyses. In the master cohort, the corresponding proportions were 72.1% for face-to-face contact nominations and 63.0% for emotional support nominations (see also online appendix table A1).

Separate DyNAMs were estimated for (a) personal face-to-face contact and (b) received emotional social support. Baseline mental health measures were included as time-invariant actor-level covariates. In the directed support models, symptom measures entered as ego effects in the rate model, capturing whether individuals with different mental health profiles differed in their propensity to become receivers of emotional social support. In the choice component, alter effects tested whether individuals with specific symptom profiles were more or less likely to be selected as support providers, while similarity effects assessed whether support exchanges were more likely between individuals with similar levels of depressive symptoms, anxiety symptoms, or ADHD symptoms.

For undirected face-to-face interaction models, mental health measures entered the coordination component through alter and similarity effects. Alter effects captured whether individuals with elevated symptom levels were generally more or less likely to be involved in face-to-face contact, whereas similarity effects assessed whether interaction events were more likely between individuals with comparable mental health profiles, capturing symptom-based homophily in everyday interaction. Similarity effects were based on dyadic similarity scores derived from the absolute difference in symptom scores, with higher values indicating greater similarity.

To facilitate interpretation of the DyNAM specifications, Table 1 provides a schematic overview of the model effects included in the analyses and illustrates how a positive coefficient should be interpreted for received emotional support and face-to-face contact, respectively. Because the two outcomes are represented by different event processes, some effects are specific to the directed support model, whereas others have corresponding interpretations in the undirected coordination model.

**Table 1:** Schematic overview of DyNAM effects for received emotional support and personal face-to-face contact

Effect	Schematic	Interpretation of positive coefficient in Models of received emotional social support	Interpretation of positive coefficient in Models of personal face-to-face contact
Ego attribute	● → event ○ event	Participants with higher values on the attribute are more likely to become involved as receivers of emotional support.	Not applicable; the coordination model does not distinguish an ego who initiates the event.
Alter attribute	i →● i ○	Potential support providers with higher values on the attribute are more likely to be selected as providers of emotional support.	Dyads involving an actor with higher values on the attribute are more likely to experience a face-to-face contact event.
Similarity	**● — ●** vs. **● — ○**	Emotional support is more likely between participants who are more similar on the attribute.	Face-to-face contact is more likely between participants who are more similar on the attribute.
Same sex	♀ — ♀ ♂ — ♂ vs. ♀ — ♂	Emotional support is more likely within same-sex dyads than within mixed-sex dyads.	Face-to-face contact is more likely within same-sex dyads than within mixed-sex dyads.
Inertia	i → j ⟶ i → j	A previous support event involving the same receiver–provider configuration increases the likelihood that this support relation occurs again.	A previous face-to-face contact between the same two actors increases the likelihood that the dyad interacts again.
Reciprocity	i → j j → i	A previous support event in one direction increases the likelihood of a subsequent support event with receiver and provider roles reversed.	Not applicable because face-to-face contact is modelled as an undirected coordination event.
Transitivity	i → k, k → j ⟶ i → j	A support event is more likely when the focal dyad is embedded in a prior two-step support path.	i — k, k — j — i — j: Face-to-face contact is more likely between actors who share a prior interaction partner.

Note 1. Black and white nodes indicate actors with relatively higher versus lower/different values on the respective attribute. For similarity effects, two matching node colours represent greater similarity, whereas differently coloured nodes represent lower similarity. Female (♀) and male (♂) symbols illustrate the same-sex effect. Arrows denote directed emotional-support events; lines denote undirected face-to-face contact events

Note 2: Key modelling assumptions. Interaction events are modelled conditional on the preceding observed event history and the set of eligible actors within the bounded cohort network. Baseline mental health characteristics are treated as time-invariant actor attributes. Support events are directed because receiver and provider roles are distinguishable, whereas face-to-face contacts are treated as undirected coordination events because the initiator cannot be identified

Models were estimated separately for the bachelor and master cohorts to respect differences in network boundaries and social context. By analysing directed and undirected DyNAM specifications, the study captures both asymmetric support processes and reciprocal everyday interaction dynamics, allowing a more differentiated exploratory analysis of how mental health symptom levels are associated with social interaction in daily life. The analyses were exploratory and process-oriented. They were designed to examine how baseline symptom levels were associated with positions in unfolding interaction and support processes, rather than to test causal effects of mental health symptoms on social behaviour.

## Results

### Descriptive characteristics of mental health and interaction patterns

Table 2 presents descriptive statistics for the bachelor and master cohorts. The bachelor cohort consisted predominantly of women (77.3%), whereas the gender distribution in the master cohort was substantially more balanced (50.0% women).

**Table 2:** Descriptive statistics for mental health symptoms and interaction measures by cohort

	Bachelor				Master			
	Mean	SD	Min	Max	Mean	SD	Min	Max
Gender (women)	0.773	0.428	0.000	1.000	0.500	0.519	0.000	1.000
Depression (PHQ-9)	8.875	3.505	3.000	17.000	9.000	4.536	3.000	16.000
Anxiety (GAD-7)	7.583	3.574	1.000	15.000	6.867	4.612	1.000	17.000
Adhd (ASRS V1.1)	9.208	3.489	1.000	17.000	9.400	4.983	4.000	18.000
Personal contact: indegree	6.021	5.582	0.000	23.000	6.874	6.662	0.000	22.000
Personal contact: outdegree	6.266	5.722	0.000	19.000	7.028	6.132	0.000	17.000
Received support: indegree	4.161	5.489	0.000	26.000	2.857	5.265	0.000	22.000
Received support: outdegree	4.073	5.757	0.000	32.000	3.143	4.533	0.000	18.000

Note. Gender was coded as 1 = woman and 0 = man. One participant in the bachelor and one in the master cohort reported a gender category outside the woman/man binary. To avoid reporting a single-cell category and because gender-related model terms were specified as binary covariates, this response was treated as missing for the descriptive gender variable and for gender-related model effects

Across both cohorts, participants reported substantial variation in mental health symptoms. In the bachelor cohort, depressive symptoms measured with the PHQ-9 averaged 8.88 (SD = 3.51), with scores ranging from 1 to 17. In the master cohort, the mean PHQ-9 score was 9.00 (SD = 4.54, range = 3–16). Anxiety symptoms measured with the GAD-7 were also somewhat higher among bachelor students (M = 7.583, SD = 3.57, range = 1–15) than among master students (M = 6.87, SD = 4.61, range = 1–17). ADHD symptoms assessed with the ASRS v1.1 were relatively similar across cohorts, with bachelor students reporting a mean score of 9.21 (SD = 3.49, range = 1–17) and master students a mean score of 9.40 (SD = 4.98, range = 4–18). Overall, the descriptive statistics indicate considerable between-person heterogeneity in all three mental health dimensions.

To contextualize symptom levels, we additionally examined the proportion of participants scoring at or above established screening thresholds. 30.4% of bachelor students (*n* = 7) and 33.3% of master students (*n* = 5) scored at or above the PHQ-9 threshold of 10. The corresponding proportions for the GAD-7 threshold of 10 were 30.4% (*n* = 7) and 33.3% (*n* = 5), respectively. For ADHD symptoms, 13.0% of bachelor students (*n* = 3) and 33.3% of master students (*n* = 5) scored at or above the recommended ASRS screening threshold of 14. These thresholds are intended for screening purposes and should not be interpreted as clinical diagnoses.

Substantial variability was also observed in everyday interaction behaviour. In the bachelor cohort, participants reported on average 6.02 incoming personal contact nominations (SD = 5.58, range = 0–23) and 6.27 outgoing contact nominations (SD = 5.72, range = 0–19). In the master cohort, the corresponding values were slightly higher, with a mean indegree of 6.87 (SD = 6.66, range = 0–22) and a mean outdegree of 7.03 (SD = 6.13, range = 0–17). The relatively large standard deviations and broad ranges indicate substantial heterogeneity in face-to-face interaction involvement within both cohorts.

Emotional support exchanges occurred less frequently than general face-to-face contact. In the bachelor cohort, received emotional support nominations averaged 4.16 incoming nominations (SD = 5.49, range = 0–26) and 4.07 outgoing nominations (SD = 5.76, range = 0–32). In the master cohort, average levels of received support were lower, with a mean indegree of 2.86 (SD = 5.27, range = 0–22) and a mean outdegree of 3.14 (SD = 4.53, range = 0–18).

Plots of individual trajectories across the 21 EMA assessments showed substantial within-person and between-person variation in both received emotional support and face-to-face contact across the study period (Online Appendix Figure A1).

### Dynamic Network Actor Models of received emotional social support

Table 3 presents the DyNAM results for received emotional social support separately for the bachelor and master cohorts. The directed support models distinguish between a rate component capturing actors’ propensity to become involved as receivers of emotional support and a choice component capturing which specific support providers are selected conditional on support exchange occurring.

**Table 3:** Dynamic Network Actor Models (DyNAMs) of received emotional social support

Rate model	Bachelor		Master	
	Effect	s.e.	Effect	s.e.
Ego sex(women)	0.829**	0.355	-0.076	0.427
Ego depressive symptoms	-0.091**	0.037	0.063	0.077
Ego anxiety	0.229**	0.037	0.230**	0.058
Ego adhd	-0.088**	0.035	-0.136*	0.071
Log-Likelihood	-289.284		-105.233	
AIC	586.567		218.465	
Choice model				
Inertia	2.366**	0.399	2.405**	0.566
Reciprocity	2.187**	0.409	0.706	0.799
Transitivity	2.090**	0.547	1.192	0.893
Alter sex(women)	3.82	28.554	-1.096	0.697
Alter depressive symptoms	-0.021	0.069	-0.119	0.111
Alter anxiety	-0.037	0.068	0.355*	0.204
Alter adhd	0.098	0.062	0.029	0.147
Same sex	-3.509	28.554	0.884*	0.526
Similarity depressive symptoms	-0.075	0.069	0.050	0.101
Similarity anxiety	0.083	0.06	0.346*	0.196
Similarity adhd	-0.037	0.075	-0.225	0.142
Log-Likelihood	-136.559		-44.371	
AIC	295.117		110.741	

Note: Ego refers to the participant reporting receipt of support, whereas alter refers to the nominated support provider; p-values ** < 0.05; * < 0.1

Across both cohorts, the choice models revealed strong endogenous structuring of emotional support exchange. Inertia effects were positive and statistically significant in both the bachelor cohort (b = 2.37, SE = 0.40) and the master cohort (b = 2.41, SE = 0.57), indicating substantial persistence in support interaction patterns over time. Actors were therefore considerably more likely to repeatedly receive support from the same individuals with whom prior support exchanges had occurred. Reciprocity effects were positive in both cohorts, although statistical significance emerged only in the bachelor cohort (b = 2.19, SE = 0.41). This finding suggests that emotional support exchanges among bachelor students tended to become mutually reinforcing over time, such that actors who had previously received support from a given individual were also more likely to become involved in reciprocal support relations. Transitivity effects were likewise positive and statistically significant in the bachelor cohort (b = 2.09, SE = 0.55), indicating local clustering tendencies in support exchange structures. In the master cohort, the transitivity effect pointed in the same direction but did not reach conventional significance levels (b = 1.19, SE = 0.89).

The rate models further revealed systematic associations between mental health symptoms and the likelihood of becoming a receiver of emotional support. In the bachelor cohort, depressive symptoms were negatively associated with the probability of actors receiving emotional support (b = -0.09, SE = 0.04). Bachelor students reporting elevated depressive symptoms were therefore less likely to become recipients of emotional support interactions during everyday life. In contrast, anxiety symptoms were positively associated with the likelihood of receiving emotional support in both cohorts (bachelor: b = 0.23, SE = 0.04; master: b = 0.23, SE = 0.06). Actors with elevated anxiety symptoms were thus more likely to receive emotional support from others. ADHD symptoms showed the opposite pattern. Higher ADHD symptom levels were associated with a lower likelihood of receiving emotional support in both the bachelor cohort (b = -0.09, SE = 0.04) and the master cohort (b = -0.14, SE = 0.07).

Gender differences emerged only partially. In the bachelor cohort, women were more likely than men to become recipients of emotional support (b = 0.83, SE = 0.36), whereas no corresponding gender difference was observed in the master cohort (b = -0.08, SE = 0.43). Because the sex-related parameters in the bachelor support choice model showed large standard errors, we additionally estimated a reduced support model for the bachelor cohort excluding sex effects (see online appendix Table A2). This sensitivity analysis produced stable coefficient estimates and did not change the substantive pattern of results.

The choice models provided more limited evidence for symptom-related support partner selection processes. In the bachelor cohort, neither alter effects nor similarity effects for depressive symptoms, anxiety symptoms, or ADHD symptoms reached statistical significance, suggesting that the selection of support providers was not systematically structured by mental health symptom profiles once endogenous network dependencies were taken into account. In the master cohort, however, anxiety symptoms were associated with support provider selection in two ways. First, actors with higher anxiety symptom levels were more likely to be selected as providers of emotional support (b = 0.36, SE = 0.20). Second, support exchanges were more likely between actors with similar anxiety symptom levels (b = 0.35, SE = 0.20), indicating symptom-based homophily in emotional support processes. No corresponding similarity effects emerged for depressive symptoms or ADHD symptoms.

As a robustness check, we re-estimated the support models while accounting for pre-existing friendship relations within the cohort (Online Appendix Table A3). The central rate-model findings were largely retained. In the bachelor cohort, depressive symptoms remained negatively associated with receiving emotional support (b = -0.10, SE = 0.04), anxiety symptoms remained positively associated (b = 0.24, SE = 0.04), and ADHD symptoms remained negatively associated (b = -0.09, SE = 0.04). In the master cohort, the positive association with anxiety (b = 0.18, SE = 0.06) and the negative association with ADHD symptoms (b = -0.26, SE = 0.09) likewise remained. Baseline friendship degree was positively associated with receiving support in the master cohort (b = 0.53, SE = 0.23), but not in the bachelor cohort. The friendship-adjusted choice models were estimated with substantially less precision, with large standard errors for several parameters, including the friendship-tie effect; these partner-selection estimates should therefore be interpreted cautiously. Overall, the robustness analysis provides support for the central rate-model findings after accounting for pre-existing social embeddedness.

### Dynamic Network Actor Models of personal face-to-face contact

Table 4 presents the DyNAM results for personal face-to-face contact separately for the bachelor and master cohorts. Because face-to-face encounters were modelled as undirected coordination events, the models estimate how actor attributes, dyadic similarity, and endogenous network dependencies are associated with the likelihood that two individuals become involved in a social interaction event.

**Table 4:** Dynamic Network Actor Models (DyNAMs) of personal face-to-face contact

	Bachelor		Master	
	Effect	s.e.	Effect	s.e.
Choice model				
Inertia	1.723**	0.185	2.175**	0.428
Transitivity	0.511**	0.116	1.350**	0.323
Alter sex(women)	1.110	1.802	-0.959	0.724
Alter depressive symptoms	0.001	0.031	0.077	0.076
Alter anxiety	0.042	0.031	-0.061	0.108
Alter adhd	0.000	0.03	-0.161	0.128
Same sex	-1.025	1.755	0.633	0.491
Similarity depressive symptoms	-0.009	0.026	0.024	0.075
Similarity anxiety	0.033	0.022	-0.272**	0.120
Similarity adhd	0.035	0.03	0.224**	0.097
Log-Likelihood	-455.127		-96.893	
AIC	930.255		213.786	

Note: p-values ** < 0.05; * < 0.1

Across both cohorts, the models revealed strong endogenous structuring of everyday interaction patterns. Inertia effects were positive and statistically significant in both the bachelor cohort (b = 1.72, SE = 0.19) and the master cohort (b = 2.18, SE = 0.43). This finding indicates substantial temporal persistence in everyday social interaction, such that actors were considerably more likely to interact repeatedly with individuals with whom prior face-to-face contact had already occurred. Transitivity effects were likewise positive and statistically significant in both cohorts (bachelor: b = 0.51, SE = 0.12; master: b = 1.35, SE = 0.32), suggesting that interaction events clustered locally within the evolving interaction network. Individuals were therefore more likely to interact with actors connected through shared interaction partners, indicating the emergence of locally cohesive interaction structures in everyday social life.

In contrast to the emotional support models, the face-to-face interaction models provided little evidence that overall involvement in social contact differed systematically according to mental health symptom levels. Across both cohorts, alter effects for depressive symptoms, anxiety symptoms, and ADHD symptoms were small and statistically non-significant. Individuals with elevated symptom levels were therefore not generally more or less likely to become involved in everyday face-to-face interaction events.

Similarly, no significant gender effects emerged. Neither the likelihood of becoming involved in interaction events with women nor same-gender interaction tendencies differed significantly from zero in either cohort. Thus, once endogenous network dependencies were taken into account, everyday face-to-face interaction coordination did not appear to be systematically structured by gender composition.

More substantial evidence emerged for symptom similarity effects within the master cohort. Similarity in anxiety symptoms was negatively associated with the likelihood of interaction (b = -0.27, SE = 0.12), indicating that master students with comparable anxiety symptom levels were less likely to interact with one another. In contrast, similarity in ADHD symptoms showed a positive association with interaction probability (b = 0.22, SE = 0.10), suggesting that individuals with similar levels of ADHD symptoms were more likely to engage in face-to-face interaction. No corresponding similarity effects emerged for depressive symptoms. In the bachelor cohort, none of the symptom similarity effects reached statistical significance.

We additionally examined whether the face-to-face contact results were robust to accounting for pre-existing friendship relations (Online Appendix Table A4). Baseline friendship was strongly associated with subsequent face-to-face interaction in both the bachelor (b = 2.45, SE = 0.30) and master cohorts (b = 3.97, SE = 0.93), indicating that interaction events occurred disproportionately within pre-existing friendship dyads. Importantly, the primary finding regarding individual symptom levels remained unchanged: alter effects for depressive symptoms, anxiety symptoms, and ADHD symptoms were not statistically significant in either cohort after accounting for friendship. Thus, there was still little evidence that students with higher symptom levels were generally more or less likely to be involved in face-to-face contact. In the master cohort, the previously significant negative anxiety-similarity effect was no longer statistically significant after adjustment for friendship, while the positive ADHD-similarity effect was reduced to marginal significance. Overall, accounting for pre-existing friendship therefore reinforced the conclusion that mental health symptom levels were not systematically associated with general involvement in face-to-face interaction, while suggesting that some dyadic similarity patterns partly overlap with existing friendship structure.

## Discussion

This exploratory intensive longitudinal network study examined how mental health symptom levels are associated with everyday social interaction by modelling face-to-face contact and received emotional support as relational events unfolding over time. Three preliminary findings stand out. First, everyday interaction was strongly structured by prior interaction history. Both personal contact and received emotional support showed pronounced inertia, indicating that students repeatedly interacted with, or received support from, the same partners over time. Transitivity was also positive across both personal-contact models and in the bachelor support model, suggesting that interaction and support were embedded in locally clustered social structures. These findings are consistent with relational and network perspectives that view social life as cumulative: current interaction opportunities are shaped by previous interaction histories and by local network embeddedness [28, 29].

Second, mental health symptoms were more clearly associated with received emotional support than with general face-to-face contact. In the support models, anxiety symptoms were positively associated with becoming a receiver of emotional support in both cohorts. This finding is consistent with prior evidence linking anxiety symptoms to excessive reassurance seeking [22]. In contrast, depressive symptoms were negatively associated with receiving emotional support in the bachelor cohort, and ADHD symptoms were negatively associated with receiving support in both cohorts. These patterns suggest that symptom domains may differ in how they are associated with support mobilization. One possible interpretation is that anxiety is associated with more visible or expressed need for emotional support, whereas depressive and ADHD symptoms may be linked to reduced access to support or interaction styles that make support less likely to occur. This interpretation aligns with prior work on depressive withdrawal, interpersonal strain, and ADHD-related difficulties in interaction coordination [9, 12, 18, 25]. However, the findings for ADHD symptoms should be interpreted with particular caution. Although the ASRS v1.1 screener [35, 36] is an established instrument and the dimensional 0–24 scoring approach is recommended for research on ADHD prevalence and correlates, internal consistency was low in the bachelor cohort. This may reflect the restricted sample size, or heterogeneity in how ADHD-related behaviours were expressed in this newly formed student context. Accordingly, the ADHD effects should be viewed as preliminary and hypothesis-generating rather than as definitive evidence of ADHD-specific interaction dynamics.

Third, the personal-contact models showed little evidence that students with elevated symptom levels were generally more or less likely to be involved in face-to-face contact. Instead, personal contact was strongly structured by pre-existing relationships and by the evolving history of interaction itself. The robustness analyses showed that baseline friendship was strongly associated with subsequent face-to-face interaction in both cohorts, while the absence of alter effects for depressive symptoms, anxiety symptoms, and ADHD symptoms remained unchanged. This distinction is substantively important. It suggests that mental health symptoms may not be systematically associated with general exposure to everyday face-to-face contact in a straightforward way, once repeated contact and clustering are accounted for. One reason may be that face-to-face contact in university settings is partly structured by shared attendance in lectures, seminars, and other institutional routines. Such opportunity structures can generate repeated co-presence and interaction independently of individual symptom profiles, thereby limiting the extent to which general contact reflects purely voluntary social approach or avoidance. Rather, symptoms may matter more strongly for the emotionally meaningful subset of interaction in which support is exchanged. This supports the conceptual distinction between social exposure and support-related interaction: being in contact with others is not equivalent to receiving emotionally supportive responses [4, 42].

The similarity effects provide preliminary evidence that symptom-based selection processes may depend on context and interaction type. For received emotional support, anxiety similarity was positively associated with support exchange in the master cohort, suggesting that students with similar anxiety levels were more likely to be involved in support interactions. For personal contact, however, anxiety similarity was negatively associated with interaction in the master cohort, whereas ADHD similarity was positively associated with interaction. Moreover, the pattern of similarity effects changed when pre-existing friendship was taken into account: additional similarity associations emerged in the support models, whereas the anxiety-similarity effect in the master face-to-face model was no longer statistically significant. These findings should be interpreted cautiously given the small sample size, but they suggest that symptom similarity does not operate uniformly across domains. Similarity in one symptom dimension may facilitate emotional understanding and support exchange, while similarity in another may structure everyday contact through shared interaction styles, activities, or social niches. This pattern is consistent with broader work showing that homophily and selection processes are highly context-dependent in social networks [2, 37].

Overall, the findings contribute to research on mental health and social relationships by illustrating how intensive longitudinal network data can shift attention from static connectedness to interaction dynamics. Rather than asking only whether individuals with mental health symptoms have fewer ties or lower perceived support, the present study shows how symptoms are associated with specific positions in unfolding interaction processes: who receives support, who is selected as a support provider, and which dyads become involved in repeated contact. This event-based perspective is particularly relevant for mental health research because social support is not only a stable resource but also an episodic process that is mobilized, withheld, or reciprocated in concrete situations [4, 43].

### Limitations and future research

Several limitations should be considered when interpreting the findings. The study was designed as an intensive longitudinal network study of everyday interaction processes rather than as a population-based study of mental health prevalence or average social connectedness. Its strength therefore lies in modelling temporally ordered micro-dynamics of interaction and support within bounded peer contexts. This design logic necessarily differs from that of large-scale survey studies: it offers detailed information on interaction sequences but only within a limited number of actors. The findings should therefore be interpreted as exploratory and hypothesis-generating evidence on interaction processes, not as population-level estimates. Future research should examine whether the observed symptom-specific patterns replicate in larger student networks, across different institutional settings, and over longer observation periods.

Second, the analytical networks cover only interactions between participating students. Participants could nominate eligible cohort members who did not participate in the study, but these events could not be included in the DyNAM analyses because mental health measures and actor-level covariates were unavailable for non-participating alters. This restriction was necessary for estimating ego, alter, and similarity effects, but it means that the models capture interaction dynamics within the observed participant networks rather than the complete cohort-wide interaction structure. The majority of nominations, however, referred to participating students and could therefore be retained, suggesting that the analytical networks captured a substantial share of reported within-cohort interaction. Nevertheless, this remains a relevant limitation because social network analyses are sensitive to boundary specification and missing actors; omitted nodes may affect estimates of clustering, reciprocity, and homophily if missingness is socially patterned [44, 45].

Third, the interaction data were collected through EMA reporting intervals rather than exact continuous-time observation. Participants reported interactions that occurred since the previous prompt, and event times were operationalized using the submission timestamp of the corresponding survey. The resulting event streams therefore approximate interaction dynamics across successive EMA windows rather than capturing the exact moment-to-moment order of all interactions. This trade-off reflects a broader tension in studying everyday social behaviour: EMA improves ecological validity and reduces reliance on global retrospective reports, but it does not provide the temporal precision of continuous passive sensing. Future studies could combine event-contingent reporting, EMA, and passive sensing to obtain more precise timestamps while retaining information on the perceived meaning and content of interaction.

Fourth, a further limitation concerns causal direction. Although mental health symptoms were assessed before the EMA period and therefore temporally preceded the interaction events analysed here, this temporal ordering does not establish that symptom differences caused subsequent interaction patterns. Prior social experiences may already have contributed to participants’ baseline mental health, and unobserved individual or contextual factors may influence both mental health and social interaction. Moreover, because mental health was assessed only at baseline, the present design cannot examine whether everyday interaction experiences subsequently contribute to changes in mental health. Repeated assessments of both mental health and social interaction would be required to investigate such reciprocal processes more directly.

Fifth, the three symptom dimensions also overlap conceptually and empirically [13, 14]. Their simultaneous inclusion helps distinguish conditional patterns of association, but shared variance between symptom domains may reduce the precision of individual coefficients, particularly in the present small samples, and limits disorder-specific interpretations.

Finally, the study relies on self-reported interaction and support nominations. Compared with retrospective global network measures, EMA reduces recall bias by shortening the reporting window [46], but reports may still be affected by memory, interpretation, motivation, and symptom-related perception. This issue is especially relevant in mental health research because depressive and anxiety symptoms may influence how individuals interpret, remember, or label social encounters and support exchanges [8, 10]. At the same time, passive sensing data alone cannot distinguish supportive, neutral, or stressful interactions. A promising direction is therefore to combine self-reported EMA, sociometric rosters, and passive behavioural traces. Such multimethod designs would allow researchers to separate physical co-presence from perceived interaction quality and support content, addressing limitations of both self-report and sensor-only approaches [26, 27].

## Conclusion

This exploratory intensive longitudinal network study examined how mental health symptom levels are embedded in the unfolding micro-dynamics of everyday social interaction among university students. By combining experience sampling with sociometric roster data and Dynamic Network Actor Models, we moved beyond static measures of social connectedness and modelled social interaction as a sequence of relational events. This approach allowed us to distinguish between two analytically and substantively different forms of everyday social life: undirected face-to-face contact and directed emotional support exchange.

The findings provide preliminary evidence that symptom levels were more consistently associated with emotional support exchange than with general face-to-face contact in university students. Anxiety symptoms were linked to a higher likelihood of receiving emotional support, whereas depressive symptoms and ADHD symptoms were associated with reduced receipt of support in at least one cohort. In contrast, general face-to-face contact was shaped primarily by endogenous network dynamics and pre-existing friendship relations, with limited evidence of associations between symptom levels and overall involvement in everyday contact.

These findings underscore the importance of distinguishing social exposure from emotionally meaningful support. Mental health symptoms may not be uniformly associated with lower or higher levels of social interaction; rather, they appear to be differentially associated with specific forms of interaction. Anxiety symptoms were associated with greater support receipt, whereas depressive and ADHD symptoms were associated with reduced support receipt in at least one cohort. At the same time, the strong role of pre-existing friendship and prior interaction patterns suggests that mental health-related interaction patterns unfold within already structured social environments, where prior contacts and local network clustering shape future opportunities for connection and support.

Overall, the study contributes to research on mental health and social relationships by showing that associations between mental health symptoms and social interaction can be better understood when interaction is treated as a dynamic process rather than as a static tie. This perspective also has practical implications for university mental health policy. Interventions should not only aim to increase students’ general social connectedness, but also attend to the concrete interaction contexts in which support is mobilized or missed. Structured opportunities for repeated low-threshold contact, peer-support formats, and early-semester integration activities may be especially relevant because everyday interaction patterns appear to become stabilized through prior contact and local clustering. If replicated in larger samples, these patterns would suggest that students with depressive or ADHD symptoms may benefit from more proactive support pathways, as they may be less likely to receive emotional support in everyday peer networks. Future research should build on this event-based perspective using larger networks, longer observation periods, and repeated mental health assessments to test whether these preliminary patterns generalize across settings, whether mental health and everyday interaction dynamics mutually reinforce one another over time, and how institutional interventions can alter these trajectories.

## Supplementary Information

Below is the link to the electronic supplementary material.

Supplementary Material 1

## Data Availability

The datasets generated and analyzed during the current study are not publicly available due to data protection requirements, the sensitive nature of the data, and the terms of informed consent provided to participants. The data contain intensive longitudinal social network information from small bounded student cohorts, including time-stamped interaction and support nominations, which may create a risk of re-identification even after removal of direct identifiers. Access to selected, de-identified materials may be considered by the corresponding author upon reasonable request, subject to applicable data protection regulations, ethical approval, and institutional data-sharing requirements.
