# Supplementary material for: Mental health symptoms and everyday social interaction among university students: an exploratory intensive longitudinal study of relational events within bounded peer networks
Source: BMC Psychol. 2026 Oct 15;14:1487. doi: 10.1186/s40359-026-05805-7 (PMC13670546; doi:10.1186/s40359-026-05805-7)
Supplement: Supplementary file 1 [file 40359_2026_5805_MOESM1_ESM.docx]

**Online Appendix**

**Tables**

Table A1. Study participation, EMA compliance, and event retention by cohort

|  | Bachelor cohort (1st semester) | | Master cohort (1st – 6th semester) | |
| --- | --- | --- | --- | --- |
|  | n | % | n | % |
| Students enrolled in the eligible study programme | 56 | 100.0 | 51 | 100.0 |
| Students participating in the PeerSmart Network Study and EMA phase | 23 | 41.1 | 15 | 29.4 |
| EMA surveys submitted | 371 | 76.8 | 286 | 90.8 |
| Reported received emotional support events | 110 | 100.0 | 73 | 100.0 |
| Received emotional support events retained for DyNAM analysis | 100 | 90.9 | 46 | 63.0 |
| Reported face-to-face interaction events | 170 | 100.0 | 147 | 100.0 |
| Face-to-face interaction events retained for DyNAM analysis | 144 | 84.7 | 106 | 72.1 |

Note. Participation rates refer to the number of students enrolled in the eligible study programme. EMA compliance refers to the proportion of submitted surveys out of all scheduled surveys during the seven-day assessment period. Event retention rates refer to the proportion of reported events involving another study participant and therefore available for DyNAM analysis.

Table A2: Dynamic Network Actor Models (DyNAMs) of received emotional social support in the bachelor cohort excluding sex effects

|  |  | Bachelor | |  |
| --- | --- | --- | --- | --- |
|  |  | Effect | s.e. |  |
| Rate model |  |  |  |  |
| Ego depressive symptoms |  | -0,111** | 0,037 |  |
| Ego anxiety |  | 0,245** | 0,037 |  |
| Ego adhd |  | -0,087** | 0,033 |  |
| Log-Likelihood |  | -292,645 |  |  |
| AIC |  | 591,289 |  |  |
|  |  |  |  |  |
| Choice model |  |  |  |  |
| Inertia |  | 2,374** | 0,4 |  |
| Reciprocity |  | 2,196** | 0,412 |  |
| Transitivity |  | 2,127** | 0,549 |  |
| Alter depressive symptoms |  | -0,012 | 0,069 |  |
| Alter anxiety |  | -0,027 | 0,067 |  |
| Alter adhd |  | 0,089 | 0,058 |  |
| Similarity depressive symptoms |  | -0,064 | 0,066 |  |
| Similarity anxiety |  | 0,088 | 0,059 |  |
| Similarity adhd |  | -0,033 | 0,072 |  |
| Log-Likelihood |  | -137,221 |  |  |
| AIC |  | 292,442 |  |  |

Note: Ego refers to the participant reporting receipt of support, whereas alter refers to the nominated support provider; p-values ** < 0.05; * < 0.1

Table A3: Dynamic Network Actor Models (DyNAMs) of received emotional social support, accounting for friendship ties

|  |  | Bachelor | |  | Master | |  |
| --- | --- | --- | --- | --- | --- | --- | --- |
|  |  | Effect | s.e. |  | Effect | s.e. |  |
| Rate model |  |  |  |  |  |  |  |
| Ego sex(women) |  | 0.718* | 0.401 |  | 1.836** | 0.933 |  |
| Ego depressive symptoms |  | -0.098** | 0.039 |  | 0.161* | 0.092 |  |
| Ego anxiety |  | 0.237** | 0.039 |  | 0.175** | 0.058 |  |
| Ego adhd |  | -0.094** | 0.037 |  | -0.259** | 0.089 |  |
| Friendship outdeg |  | 0.056 | 0.094 |  | 0.534** | 0.231 |  |
| Log-Likelihood |  | -289.107 |  |  | -101.555 |  |  |
| AIC |  | 178.506 |  |  | 51.416 |  |  |
|  |  |  |  |  |  |  |  |
| Choice model |  |  |  |  |  |  |  |
| Inertia |  | 0.096 | 0.415 |  | -3.194** | 1.608 |  |
| Reciprocity |  | 0.009 | 0.422 |  | 1.23 | 1.187 |  |
| Transitivity |  | 0.267 | 0.542 |  | -0.16 | 1.237 |  |
| Friendship tie |  | 15.291 | 66.227 |  | 39.68 | 179.633 |  |
| Alter sex(women) |  | 0.066 | 238.796 |  | -6.375 | 62.487 |  |
| Alter depressive symptoms |  | -0.014 | 0.09 |  | 0.268 | 0.254 |  |
| Alter anxiety |  | -0.023 | 0.075 |  | 0.417 | 0.603 |  |
| Alter adhd |  | 0.22* | 0.121 |  | -0.531 | 0.379 |  |
| Same sex |  | -0.748 | 238.795 |  | 6.47 | 62.473 |  |
| Similarity depressive symptoms |  | -0.019 | 0.09 |  | -0.305 | 0.299 |  |
| Similarity anxiety |  | 0.131** | 0.058 |  | 3.051** | 1.227 |  |
| Similarity adhd |  | 0.181 | 0.134 |  | -1.082** | 0.52 |  |
| Log-Likelihood |  | -77.253 |  |  | -13.708 |  |  |
| AIC |  | 178.506 |  |  | 51.416 |  |  |

*Note 1: p-values ** < 0.05; * < 0.1
Note 2: Friendship was assessed at baseline using a roster-based nomination question asking participants to identify those cohort members they considered friends. For the present analyses, friendship was operationalized as an undirected dyadic indicator coded 1 if either member of a dyad nominated the other as a friend and 0 otherwise.*

Table A4: Dynamic Network Actor Models (DyNAMs) of personal face-to-face contact, accounting for friendship ties

|  |  | Bachelor | |  | Master | |  |
| --- | --- | --- | --- | --- | --- | --- | --- |
|  |  | Effect | s.e. |  | Effect | s.e. |  |
| Choice model |  |  |  |  |  |  |  |
| Inertia |  | 0.087 | 0.227 |  | 0.400 | 0.567 |  |
| Transitivity |  | 0.219* | 0.128 |  | 0.473 | 0.354 |  |
| Friendship tie |  | 2.451** | 0.295 |  | 3.965** | 0.925 |  |
| Alter sex(women) |  | 0.680 | 1.200 |  | -0.571 | 0.820 |  |
| Alter depressive symptoms |  | 0.001 | 0.033 |  | 0.155* | 0.089 |  |
| Alter anxiety |  | 0.041 | 0.032 |  | -0.12 | 0.149 |  |
| Alter adhd |  | 0.039 | 0.034 |  | -0.178 | 0.135 |  |
| Same sex |  | -0.698 | 1.142 |  | 0.147 | 0.633 |  |
| Similarity depressive symptoms |  | -0.013 | 0.028 |  | -0.012 | 0.094 |  |
| Similarity anxiety |  | 0.016 | 0.023 |  | -0.081 | 0.150 |  |
| Similarity adhd |  | 0.077** | 0.034 |  | 0.197* | 0.110 |  |
| Log-Likelihood |  | -402.229 |  |  | -82.229 |  |  |
| AIC |  | 826.458 |  |  | 186.458 |  |  |

*Note1: p-values ** < 0.05; * < 0.1*

*Note 2: Friendship was assessed at baseline using a roster-based nomination question asking participants to identify those cohort members they considered friends. For the present analyses, friendship was operationalized as an undirected dyadic indicator coded 1 if either member of a dyad nominated the other as a friend and 0 otherwise.*

**Figures**

Figure A1. Individual trajectories of reported social interaction events across the EMA study period.

*Note. Panels show the number of interaction partners observed for each participant at each of the 21 EMA assessments. Lines connect consecutive observed assessments; gaps indicate assessments without available information. Only interactions involving members of the respective analytical cohort network are included.*
